# Identification and Phylogenetic Analysis of the Complete Chloroplast Genomes of Three* Ephedra* Herbs Containing Ephedrine

**DOI:** 10.1155/2019/5921725

**Published:** 2019-03-03

**Authors:** Xinlian Chen, Yingxian Cui, Liping Nie, Haoyu Hu, Zhichao Xu, Wei Sun, Ting Gao, Jingyuan Song, Hui Yao

**Affiliations:** ^1^Engineering Research Center of Tradition Chinese Medicine Resource, Ministry of Education, Institute of Medicinal Plant Development, Chinese Academy of Medical Sciences & Peking Union Medical College, Beijing 100193, China; ^2^Institute of Chinese Materia Medica, China Academy of Chinese Medicinal Sciences, Beijing 100700, China; ^3^Key Laboratory of Plant Biotechnology in Universities of Shandong Province, College of Life Sciences, Qingdao Agricultural University, Qingdao 266109, China

## Abstract

Ephedrae Herba and Ephedrae Radix et Rhizoma (Mahuang) have been used as Chinese herbal medicines.* Ephedra *plants mainly live in deserts and have good governance of desertification. Despite their important medicinal and environmental protection value, dietary supplements containing ephedrine from* Ephedra *species may threaten the health of people. Morphological resemblance amongst species causes difficulty in identifying the original species of* Ephedra *herbs. Chloroplast (CP) genome shows good prospects in identification and phylogenetic analysis. This study introduced the structures of the CP genomes of three* Ephedra *species and analysed their phylogenetic relationships. Three complete CP genomes of* Ephedra *showed four-part annular structures, namely, two single-copy regions and two inverted repeat regions. The entire CP genomes of three* Ephedra *species in terms of size were 109,550 bp (*E. sinica*), 109,667 bp (*E. intermedia*), and 109,558 bp (*E. equisetina*). Each CP genome of the three* Ephedra *species encoded 118 genes, including 73 protein-coding genes, 37 tRNA genes and 8 ribosomal RNA genes. Eleven high-variation regions were screened through mVISTA to be potential specific DNA barcodes for identifying* Ephedra *species. Maximum likelihood and maximum parsimony trees showed that CP genomes could be used to identify* Ephedra *species. The* Ephedra *species had a close phylogenetic relationship with* Gnetum *species and* Welwitschia mirabilis*. This research provided valuable information for the identification and phylogenetic analysis of gymnosperms and drug safety of* Ephedra*.

## 1. Introduction

Ephedrae Herba (Mahuang), as a Chinese herbal medicine, is the dry grassy stem of* Ephedra sinica *Stapf,* E. intermedia *Schrenk et C. A. Mey. or* E. equisetina *Bge. It is used for perspiratory, antitussive, antipyretic, and anti-inflammatory purposes [1]. It has also been utilised for more than 2500 years [2, 3]. It is also applied to Kampo medicine in Japan [4]. Similarly, the Chinese herbal medicine Ephedrae Radix et Rhizoma comes from the dry roots and rhizomes of* E. sinica *or* E. intermedia *[1]. It is an antiperspirant and used for spontaneous sweating and night sweat.* Ephedra *(Ephedraceae) belongs to Gymnospermae and comprises approximately 40 known species. They are distributed in arid and desert regions ranging from Asia and Southeastern Europe to Northern Africa and America [5]. Their unique habitat indicates that they have powerful resistance to drought, cold, and sand burial and are commonly used as a sand binder [5, 6].

Ephedrae Herba contains ephedrine [7], pseudoephedrine [8], norpseudoephedrine [9], and methylephedrine [10, 11], which are abundant in the three* Ephedra *plants and have pharmaceutically active anodyne and antifebrile effects [12]. The highest alkaloid content is found in* E. equisetina *followed by* E. sinica *and* E. intermedia*.* E. sinica *[5, 13] and* E. equisetina *mainly consist of ephedrine, whereas* E. intermedia *mainly contains pseudoephedrine [13–15]. In some instances,* Ephedra*-based products are used as bronchodilators in traditional Asian medicines [16]. Since the 20th century, dietary supplements containing ephedra alkaloids have been widely promoted and used in America because of their effects on weight loss and energy increase. However, these supplements may threaten the health of people [17]. The Food and Drug Administration prohibited the sale of dietary supplements containing* Ephedra *spp. or ephedrine alkaloids in April 2004 [18]. Using* Ephedra *or ephedrine and caffeine is associated with an increased risk of psychiatric, autonomic, or gastrointestinal symptoms and heart palpitations [19].

Few contrasting morphological characters are observed when* Ephedra *species do not bear flowers or seeds, thereby causing difficulty in identifying the original species of* Ephedra *Herb. This genus has also been systematically studied [20]. Different* Ephedra *species, habitats, and picking times can be distinguished by diffuse reflectance Fourier transform near infrared spectroscopy [21].* Ephedra *species has been identified, and their phylogenetic relationship has been reconstructed through chloroplast and nuclear DNA sequences. Results have been applied to identify crude drugs obtained in the Chinese market [20, 22, 23]. ITS2 sequence shows a sufficient resolution amongst Ephedrae Herba and its closely related species but fails to distinguish amongst three original Ephedrae Herba species (*E. sinica*,* E. intermedia, *and* E. equisetina*) [24].

Chloroplast plays an important role in photosynthesis, transcription, or translation [25]. As one of the three genomes of plants, CP genome shows good potential for species identification and phylogenetic reconstruction [26–28]. Studies have aimed to use the entire CP genome as a super barcode for species identification [29–31]. In this study, the structures of the CP genomes of the three* Ephedra *species were introduced in the Chinese Pharmacopoeia, and the identification ability of the CP genomes on this genus was analysed. This study provided invaluable information for studies on gymnosperm identification and phylogenetic analysis.

## 2. Materials and Methods

### 2.1. DNA Sources

Fresh* E. intermedia *stems were collected from Altay Prefecture, Xinjiang Uygur Autonomous Region, China. Fresh* E. sinica *and* E. equisetina *stems were obtained from Beijing Medicinal Plant Garden, The Institute of Medicinal Plant Development (IMPLAD), Chinese Academy of Medical Sciences. These three species were identified by Prof. Yulin Lin from IMPLAD. Voucher specimens were deposited in the herbarium at IMPLAD.

### 2.2. DNA Sequencing, Assembly and Annotation

Total DNA was extracted with a DNeasy Plant Mini Kit (Qiagen Co., Germany). Shotgun libraries with insert sizes of 500 bp were built. Total DNA was sequenced in Illumina HiSeq X. The libraries were sequenced on an Illumina HiSeq X platform to produce 150 bp paired-end reads. Low-quality reads and adapters were filtered from the raw data by using Trimmomatic [32]. Then, the remaining clean reads were used to assemble the CP genome sequences. The CP sequences of all plants downloaded from the National Center for Biotechnology Information (NCBI) constituted the reference database. Subsequently, the clean sequences were mapped to the database, and the mapped reads were extracted on the basis of coverage and similarity. The extracted reads were assembled into contigs by using SOAPdenovo2 [33]. The scaffold of the CP genome was constructed using SSPACE [34], and gaps were filled using GapFiller [35]. The accuracy of the assembly of the four boundaries, namely, large single-copy (LSC), small single-copy (SSC), and inverted repeat (IRa and IRb) regions, was verified by amplicons obtained from specific polymerase chain reaction primers (Table S1). The CP genomes of the three* Ephedra *species were initially annotated using the online programs Dual Organellar GenoMe Annotator [36] and CPGAVAS [37] and then manually corrected. The assembled complete CP genome sequences of the three species were submitted to NCBI with the accession numbers MH161420 (*E. equisetina*), MH161421 (*E. intermedia*), and MH161422 (*E. sinica*).

### 2.3. Genome Analysis

tRNA genes were identified with tRNAscan-SE [38]. CP genome maps were generated using Organellar Genome DRAW (OGDRAW) v1.2 [39] and then manually corrected. The GC content was calculated using MEGA 6.0 [40]. REPuter (University of Bielefeld, Bielefeld, Germany) [41] was employed to identify the size and location of repeat sequences in the CP genomes of the three* Ephedra *species. Simple sequence repeats (SSRs) were detected with MISA (http://pgrc.ipk-gatersleben.de/misa/). All of the repeated sequences were manually verified, and excess data were removed. The distribution of codon usage was estimated using MEGA 6.0 [40]. All these methods were also used in identification of* Ligularia *herbs using complete CP genome (https://www.frontiersin.org/articles/10.3389/fphar.2018.00695/full) [31].

### 2.4. Phylogenetic Analysis

The mVISTA [42] was used to compare the three* Ephedra *species and two published* Ephedra *species with* E. intermedia *as a reference genome. The nucleotide diversity of the CP genome was analysed using the sliding window method implemented in DnaSP v5.10 [43]. The step size was set to 200 bp with a window length of 800 bp. A phylogenetic tree with* Selaginella uncinata *and* Equisetum arvense *as outgroups was constructed on the basis of maximum likelihood (ML) and maximum parsimony (MP) analysis in MEGA 6.0. The details of the selected species excluding the three* Ephedra *species are presented in Table S2.

## 3. Results and Discussion

### 3.1. Molecular Features of the CP Genomes of Three* Ephedra* Species

**Figure 1:**
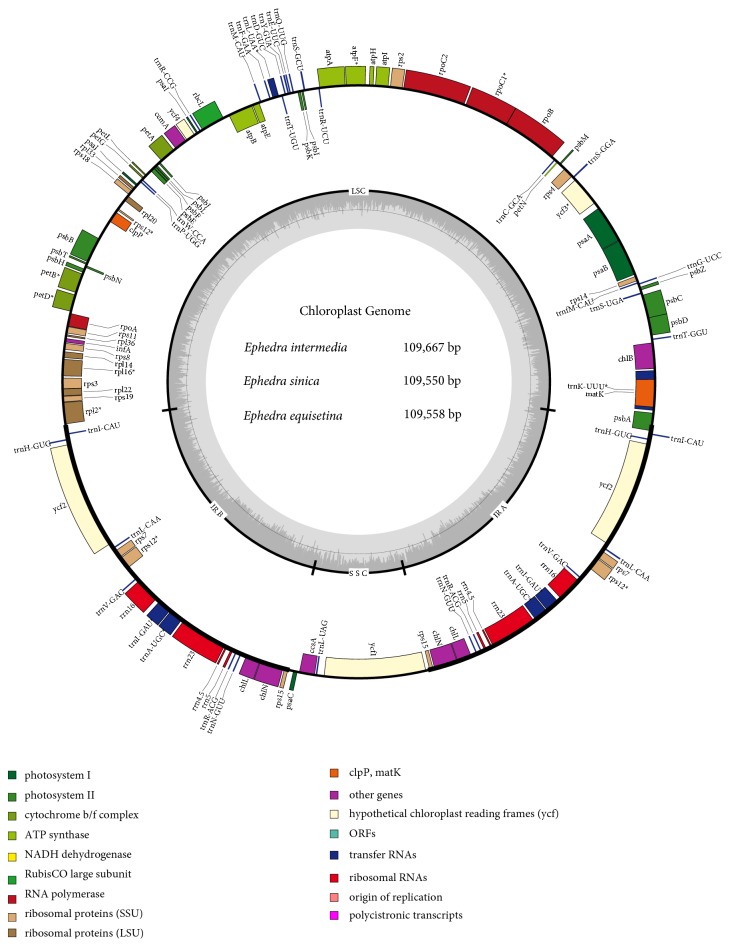
*Gene maps of the complete CP genomes of the three Ephedra species*. Genes on the inside of the circle are transcribed clockwise, while those outside are transcribed counter clockwise. The darker gray in the inner circle corresponds to GC content, whereas the lighter gray corresponds to AT content.

Three complete CP genomes of* Ephedra *showed four-part annular structures, namely, an LSC, an SSC, and two inverted repeat (IR) regions similar to most land plants (Figure 1) [44]. The lengths of the whole CP genomes were 109,667 bp (*E. intermedia*), 109,550 bp (*E. sinica*), and 109,558 bp (*E. equisetina*). The lengths of the LSC regions were 59,936 bp (*E. intermedia*), 59,961 bp (*E. sinica*), and 59,976 bp (*E. equisetina*). The lengths of the SSC regions were 8,247 bp (*E. intermedia*), 8,103 bp (*E. sinica*), and 8,078 bp (*E. equisetina*). The lengths of the IR regions were 20,742 bp (*E. intermedia*), 20,743 bp (*E. sinica*), and 20,752 bp (*E. equisetina*). The CP genomes of* Ephedra *were the most reduced and compact amongst the elucidated photosynthetic land plants, such as* Ginkgo biloba *[26],* Cycas *[45],* Gnetum *[46, 47], and* Welwitschia mirabilis *[48]. The GC contents were 36.6% (*E. intermedia *and* E. equisetina*) and 36.7% (*E. sinica*), which were lower than those of some gymnosperms [46, 47, 49]. The GC contents of the IR and LSC regions of the three species were 42% and 34.2%, respectively. The GC contents of the SSC regions were 27.3% (*E. intermedia*), 27.8% (*E. sinica*), and 27.5% (*E. equisetina*). The sizes of the four regions and the GC contents of the three* Ephedra *species were similar to previously published* E. equisetina *[48] and* E. foeminea *[50] (Table 1).

The CP genomes of the three* Ephedra *species each only had 118 genes, including 73 protein-coding genes, 37 tRNA genes and 8 rRNA genes. The CP genomes consisted of the coding regions from 73.87% (*E. intermedia*) to 73.93% (*E. equisetina*), which were greater than those of some gymnosperms, such as* W. mirabilis *[48] and* Gnetum *[46, 47]. The rates of noncoding regions, including intergenic spacers and introns, varied from 26.07% (*E. equisetina*) to 26.13% (*E. intermedia*). The genes of the CP genomes contained in the three* Ephedra *species are shown in Table 2. Furthermore, 18 duplicated genes were found in the IR regions: 6 protein-coding genes (*chlL*,* chlN*,* rps12*,* rps15*,* rps7, *and* ycf2*), 8 tRNA genes (*trnA-UGC*,* trnH-GUG*,* trnI-CAU*,* trnI-GAU*,* trnL-CAA*,* trnN-GUU*,* trnR-ACG, *and* trnV-GAC*), and 4 rRNA genes (*rrn16*,* rrn23*,* rrn4.5, *and* rrn5*).* psbA *spanned the IRb and LSC regions, and 13 genes (*atpF*,* rps7*,* petB*,* petD*,* rpl16*,* rpl2*,* rpoC1*,* trnA-UGC*,* trnT-UGU*,* trnI-GAU*,* trnK-UUU*,* trnL-CAA, *and* trnV-GAC*) consisting of only one intron were observed. In addition, 2 genes containing two introns (*rps12 *and* ycf3*) were found.* matK *was located within the intron of* trnK-UUU *in the* Ephedra *CP genomes. No NADH dehydrogenase genes were observed in the three* Ephedra *CP genomes.

**Figure 2:**
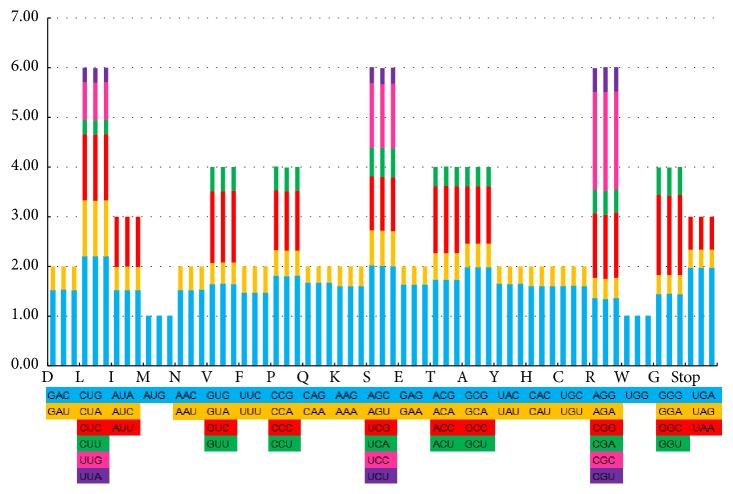
*Codon content of 20 amino acid and stop codons in all protein-coding genes of the CP genomes of three Ephedra species*. The order of every three columns is* E. intermedia*,* E. sinica, *and* E. equisetina*, respectively.

The codon content of the 20 amino acid and stop codons in all of the protein-coding genes of the CP genomes of* Ephedra *species are shown in Figure 2. The Relative Synonymous Codon Usage (RSCU) of the three* Ephedra *species is shown in Table S3. RSCU revealed a high proportion of synonymous codons having A and U in the third position in* Ephedra*. Protein-coding genes comprised 22,999 codons in* E. sinica *to 23,023 codons in* E. intermedia*. Codons for leucine, isoleucine, and lysine were the most abundant, whereas those for cysteine, tryptophane, and methionine were the least.

### 3.2. Repeat Sequences and SSRs

**Figure 3:**
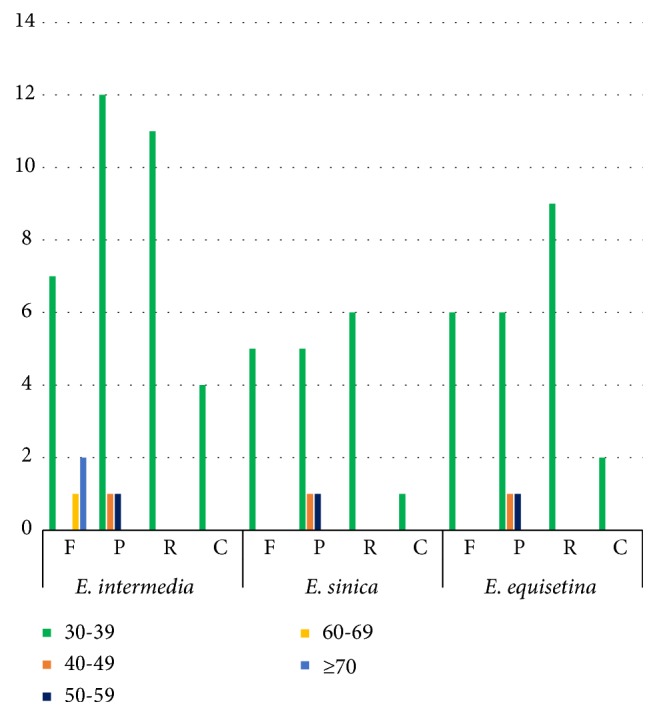
*Repeat sequences in three CP genomes*. REPuter was used to identify repeat sequences with length ≥ 30 bp and sequences identified ≥ 90% in the CP genomes. F, P, R, and C indicate the repeat types F (forward), P (palindrome), R (reverse), and C (complement). Repeats with different lengths are indicated in different colors.

Significant differences were observed in the number distribution of long repeat sequences amongst the three* Ephedra *species (Figure 3). Our results revealed 4 complement repeats, 10 forward repeats, 14 palindromic repeats, and 11 reverse repeats in the CP genome of* E. intermedia*. Furthermore, 1 complement repeat, 5 forward repeats, 7 palindromic repeats, and 6 reverse repeats were found in the CP genome of* E. sinica*. In the CP genome of* E. equisetina*, 2 complement repeats, 6 forward repeats, 8 palindromic repeats, and 9 reverse repeats were present. SSRs (1–6 nucleotide repeats) were abundant in the three* Ephedra *CP genomes. SSRs can offer relevant information for the analysis of phylogenetic relationships and population genetics [51–53]. The sequences of SSRs contained an A or T base, resulting in AT richness of the CP genome [54]. The distributions of SSRs in the three species were detected. Mononucleotide repeats A and T were the two most common types (Table S3). Few other types were observed. The MISA software identified 55 (*E. intermedia*) to 62 (*E. sinica*) SSRs in the three* Ephedra *CP genomes. Most SSRs were distributed in the LSC and SSC regions. Each species of* Ephedra *had species-specific SSRs.* E. intermedia *and* E. sinica *had one and two mononucleotide C SSRs, respectively, which were not in* E. equisetina*.* E. sinica *and* E. equisetina *contained one and two dinucleotide TA SSRs, respectively, which were not found in* E. intermedia*. Only* E. sinica *contained one tetranucleotide TTCT SSRs. Only* E. equisetina *contained one tetranucleotide CTAT SSRs. The mass variation in SSRs in the three* Ephedra *CP genomes would offer invaluable resources for the marker development and population genetics of this genus.

### 3.3. Comparative Analysis of* Ephedra* CP Genomes

**Figure 4:**
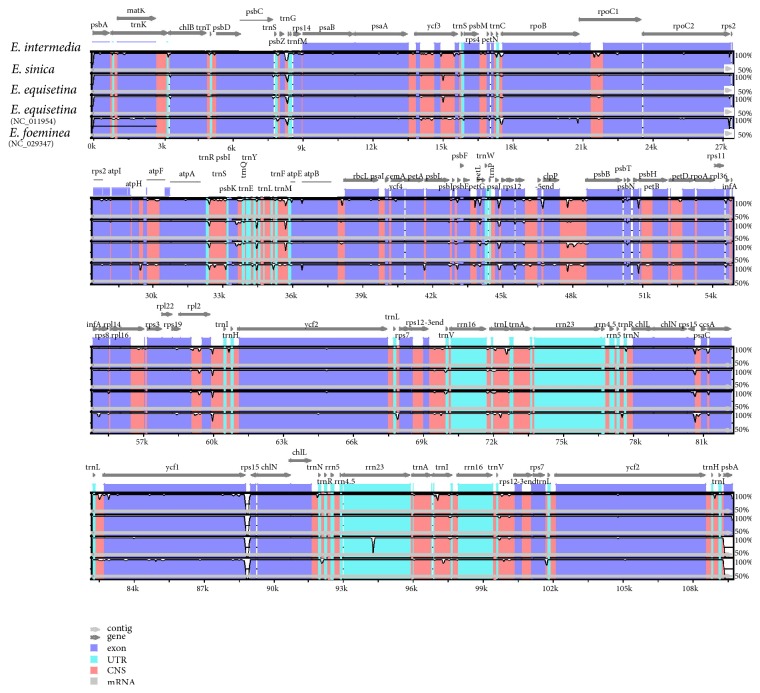
*Sequence identity plot comparing five CP genomes with E. intermedia as a reference using mVISTA*. Gray arrows and thick black lines above the alignment indicate genes with their orientation and the position of the IRs, respectively. A cutoff of 70% identity was used for the plots, and the Y-scale represents the percent identity ranging from 50 to 100%.

**Figure 5:**
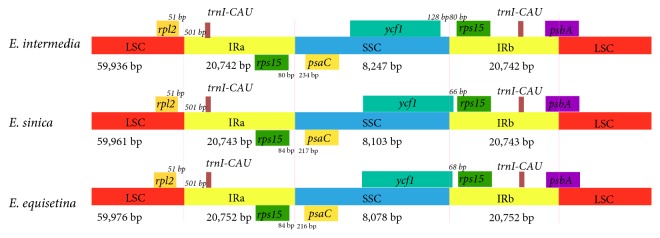
*Comparison of the borders of LSC, SSC, and IR regions amongst three CP genomes*. Number above the gene features means the distance between the ends of genes and the borders sites. These features are not to scale.

**Figure 6:**
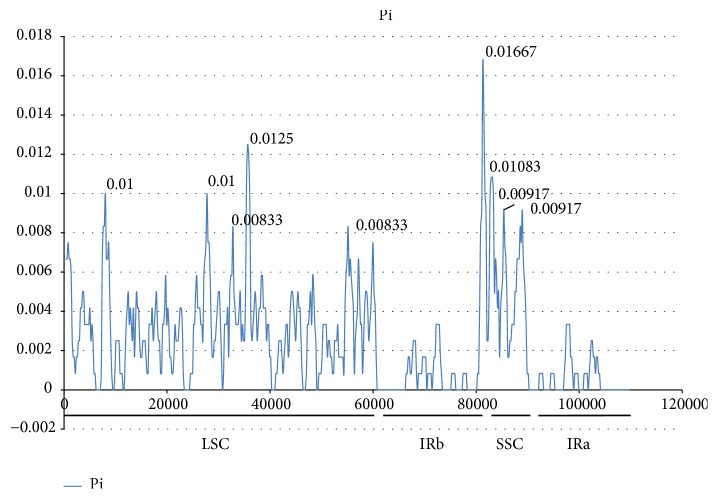
*Sliding window analysis of the whole CP genomes*. Window length: 800 bp; step size: 200 bp. X-axis: position of the midpoint of a window. Y-axis: nucleotide diversity of each window. Pi amongst three* Ephedra *species. Mutational hotspots and highly divergent loci were marked.

The annotated genes of the three studied* Ephedra *species and the two published* Ephedra *species were compared using mVISTA [42]. mVISTA (Figure 4) revealed that the CP genomes of the five* Ephedra *species showed similarity and conservatism. The divergence level of the noncoding regions was higher than that of the coding regions. The divergence level of the single-copy regions was higher than that of the IR regions. Approximately 11 high-variation regions were found in mVISTA, and they were distributed in the sequences mainly in noncoding regions, including* psbZ-trnG*,* petN-rpoB*,* trnR-trnM*,* psbJ-rpl20*,* clpP-psbB*,* rrn16-trnI*,* rps15-ccsA*,* ycf1-rps15, *and* trnV-rps12*, and in two genes, namely,* ycf3 *and* rpl2*. These sequences could provide potential information to identify* Ephedra *species. In addition, the boundaries of the four regions of the three* Ephedra *CP genomes were compared in detail (Figure 5). In the junction positions, the sites of most genes in the border region were similar. However,* ycf1 *was located entirely on the left of the SSC-IRb boundary in* E. intermedia*, whereas 18 bp was located in the IRb regions in* ycf1 *in* E. sinica *and* E. equisetina*. The average nucleotide diversity (Pi) amongst the three* Ephedra *species was 0.00252 (Figure 6). Mutational hotspots with high Pi values (>0.008) were located in the LSC and SSC regions rather than in the IR regions.

### 3.4. Identification and Phylogenetic Analysis

**Figure 7:**
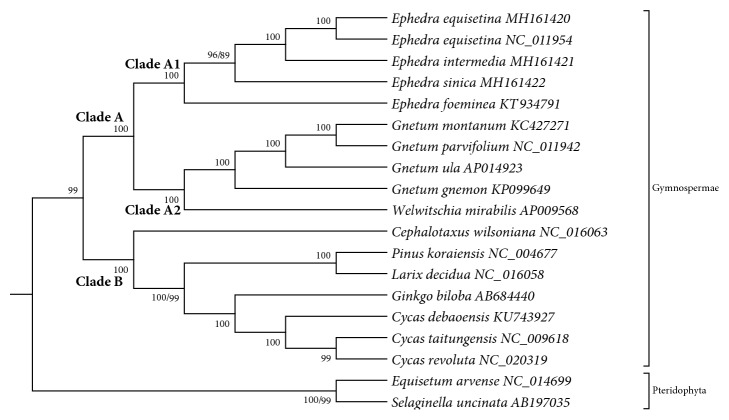
*Phylogenetic trees constructed using ML and MP methods based on common protein-coding genes of three Ephedra and other 16 species*. Numbers (MP/ML) above the branches are bootstrap support values. Only one support value means the same value.

Chloroplast genome has important implications for phylogenetic studies [28, 55]. In addition to three* Ephedra *species, 16 species were chosen to construct ML and MP trees to identify the phylogenetic position of* Ephedra *species based on 53 common protein-coding genes by using MEGA 6.0 (Figure 7). The 16 species were from Gymnospermae (14 species) and Pteridophyta (*Equisetum arvense and Selaginella uncinata *as outgroups). The alignment covered 52,722 bp. The results revealed the same tree topology of ML and MP. Each species of Gymnospermae and Pteridophyta clustered into a monophyletic group on the basis of topologic structure. Gymnospermae species were divided into two branches (Clade A and Clade B). Clade A was divided into two subbranches, namely, Clade A1 and Clade A2, with a bootstrap support value of 100%. In Clade A, Clade A1 formed a strongly supported monophyletic clade sister to Clade A2. Each branch of* Ephedra *species showed high support (bootstrap value ≥ 89%), indicating that the four* Ephedra *species could be identified. Two* E. equisetina *and* E. intermedia *clustered into a monophyletic clade, indicating their close phylogenetic relationship. Clade A2 included* Gnetum *species and* W. mirabilis*, revealing a close phylogenetic relationship with* Ephedra*. These data could be used for the identification, phylogenetic analysis, and population studies of* Ephedra *species.

## 4. Conclusions

In this study, the CP genomes of three* Ephedra *species were sequenced and analysed. The results revealed the basic structures, conservation, and variability of the sequences. Eleven variation regions were screened to be potential DNA barcodes for the identification of this genus. The ML and MP trees indicated that the CP genomes could be used to identify* Ephedra *species.* Ephedra *species showed a close phylogenetic relationship with* Gnetum *species and* W. mirabilis*. The data obtained in this study would be a helpful basis for further research involving the identification and phylogenetic analysis of gymnosperms and the safe medication of* Ephedra*.

## Figures and Tables

**Table 1:** Statistics for assembly of the three CP genomes and two published of *Ephedra *species.

Latin name	*E. intermedia*	*E. sinica*	*E. equisetina*	*E. equisetina* NC_011954	*E. foeminea* NC_029347
Gene size (bp)	109,667	109,550	109,558	109,518	109,584
LSC (bp)	59,936	59,961	59,976	59,906	60,027
SSC (bp)	8,247	8,103	8,078	8,104	8,079
IR (bp)	20,742	20,743	20,752	20,754	20,739
GC Content (%)	36.6	36.7	36.6	36.7	36.7

**Table 2:** List of genes found in the three CP genomes of *Ephedra *species.

No.	Group of genes	Gene names	Amount
1	Photosystem I	*psaA*, *psaB*, *psaC*, *psaI*, *psaJ*	5
2	Photosystem II	*psbA*, *psbB*, *psbC*, *psbD*, *psbE*, *psbF*, *psbH*, *psbI*, *psbJ*, *psbK*, *psbL*, *psbM*, *psbN*, *psbT*, *psbZ*	15
3	Cytochrome b/f complex	*petA*, *petB*^*∗*^, *petD*^*∗*^, *petG*, *petL*, *petN*	6
4	ATP synthase	*atpA*, *atpB*, *atpE*, *atpF*^*∗*^, *atpH*, *atpI*	6
5	RubisCO large subunit	*rbcL*	1
6	RNA polymerase	*rpoA*, *rpoB*, rpoC1^*∗*^, *rpoC2*	4
7	Ribosomal proteins (SSU)	*rps2*, *rps3*, *rps4*, rps7^*∗*^(×2), *rps8*, *rps11*, rps12^*∗∗*^(×2), *rps14*, *rps15(*×2), *rps18*, *rps19*	14
8	Ribosomal proteins (LSU)	rpl2^*∗*^, *rpl14*, rpl16^*∗*^, *rpl20*, *rpl22*, *rpl33*, *rpl36*	7
9	Photochlorophyllide reductase subunit B/L/N	*chlB*, *chlL*(×2), *chlN*(×2)	5
10	Proteins of unknown function	*ycf1*, *ycf2*(×2), *ycf3*^*∗∗*^ , *ycf4*	5
11	Transfer RNAs	37 *tRNA*s (6 contain an intron)	37
12	Ribosomal RNAs	*rrn4.5*(×2), *rrn5*(×2), *rrn16*(×2), *rrn23*(×2)	8
13	Other genes	*clpP*, *matK*, *ccsA*, *cemA*, *infA*	5

^*∗*^Gene contains one intron;  ^*∗∗*^gene contains two introns; (×2) indicates the number of the repeat unit is 2.

## Data Availability

The assembled complete CP genome sequences of the three species were submitted to NCBI with the accession numbers MH161420 (*E. equisetina*), MH161421 (*E. intermedia*), and MH161422 (*E. sinica*). Users could download the data as a reference for research purposes only.

## Supplementary Materials

Supplementary Material Table S1: validated primers for confirming four boundaries of the CP genomes from three* Ephedra* species. Table S2: the details of the selected species in the ML trees. Table S3: the codon usage of CP genomes of three* Ephedra *species. Table S4: SSR types and numbers of CP genomes of three* Ephedra *species.
