# Supplementary material for: Identification and Phylogenetic Analysis of the Complete Chloroplast Genomes of Three Ephedra Herbs Containing Ephedrine
Source: Biomed Res Int. 2019 Mar 3;2019:5921725. doi: 10.1155/2019/5921725 (PMC6420972; doi:10.1155/2019/5921725)
Supplement: Supplementary Material — Table S1: validated primers for confirming four boundaries of the CP genomes from three Ephedra species. Table S2: the details of the selected species in the ML trees. Table S3: the codon usage of CP genomes of three Ephedra species. Table S4: SSR types and numbers of CP genomes of three Ephedra species. [file 5921725.f1.docx]

**Identification and phylogenetic analysis of the complete chloroplast genomes of three** ***Ephedra* herbs containing ephedrine**

**supplementary files for review**

CHEN Xin-lian^1^, CUI Ying-xian^1^, NIE Li-ping^1^, HU Hao-yu^2^, XU Zhi-chao^1^, SUN Wei^2^, GAO Ting^3^, SONG Jing-yuan^1^, YAO Hui^1^*

1 Engineering Research Center of Tradition Chinese Medicine Resource, Ministry of Education, Institute of Medicinal Plant Development, Chinese Academy of Medical Sciences & Peking Union Medical College, Beijing 100193, China

2 Institute of Chinese Materia Medica, China Academy of Chinese Medicinal Sciences, Beijing 100700, China;

3 Key Laboratory of Plant Biotechnology in Universities of Shandong Province, College of Life Sciences, Qingdao Agricultural University, Qingdao 266109, China

Corresponding author: Yao Hui, scauyaoh@sina.com

Running title: Chloroplast genomes of three *Ephedra* species

Table S1 । Validated primers for confirming four boundaries of the CP genomes from three *Ephedra* species

| Regions | Forward/Reverse | Base composition of primers (5' to 3') | Number of bases |
| --- | --- | --- | --- |
| IRb-LSC | Y90-IL-F | ACCTCCTTTAGAGGTTTGGTACG | 23 |
|  | Y90-IL-R | GGGCCGTGAATGGGAACTTA | 20 |
| LSC-IRa | Y90-LI-F | GCCTAACCGGTCTTTCCGAT | 20 |
|  | Y90-LI-R | ACCTCCTTTAGAGGTTTGGTACG | 23 |
| IRa-SSC | Y90-IS-F | AGATCGGGCTACTCTGGTGA | 20 |
|  | Y90-IS-R | TGTTGGACAAGCAGATTCACA | 21 |
| SSC-IRb | Y90-SI-F | CGGGAAACTAAGTAAGGCAAAACT | 24 |
|  | Y90-SI-R | TCGGGCTACTCTGGTGAGAA | 20 |
| IRb-LSC | Y91-IL-F | ACCTCCTTTAGAGGTTTGGTACG | 23 |
|  | Y91-IL-R | GGGCCGTGAATGGGAACTTA | 20 |
| LSC-IRa | Y91-LI-F | GGGCCTAACCGGTCTTTCC | 19 |
|  | Y91-LI-R | CCTCCTTTAGAGGTTTGGTACG | 22 |
| IRa-SSC | Y91-IS-F | TCGGGCTACTCTGGTGAGAA | 20 |
|  | Y91-IS-R | TTCTGTTCGTGGAGCAGAGG | 20 |
| SSC-IRb | Y91-SI-F | GTCGAATTGCCAAAAATAACAGGG | 24 |
|  | Y91-SI-R | GATCGGGCTACTCTGGTGAG | 20 |
| IRb-LSC | Y92-IL-F | ACCTCCTTTAGAGGTTTGGTACG | 23 |
|  | Y92-IL-R | GGGCCGTGAATGGGAACTTA | 20 |
| LSC-IRa | Y92-LI-F | GCCTAACCGGTCTTTCCGAT | 20 |
|  | Y92-LI-R | ACCTCCTTTAGAGGTTTGGTACG | 23 |
| IRa-SSC | Y92-IS-F | ATCGGGCTACTCTGGTGAGA | 20 |
|  | Y92-IS-R | CTTACAACCGGTCCAAGGGA | 20 |
| SSC-IRb | Y92-SI-F | GTCGAATTGCCAAAAATAACAGGG | 24 |
|  | Y92-SI-R | GATCGGGCTACTCTGGTGAG | 20 |

“Y90” stands for “*Ephedra intermedia*”, “Y91” stands for “*Ephedra sinica*”, “Y92” stands for “*Ephedra equisetina*”.

Table S2 । The details of the selected species in the ML trees

| 序号 | Latin name | Accession number | Length of CP genome |
| --- | --- | --- | --- |
| 1 | *Equisetum arvense* | NC_014699 | 133,309 |
| 2 | *Ginkgo biloba* | AB684440 | 156,945 |
| 3 | *Cycas taitungensis* | NC_009618 | 163,403 |
| 4 | *Cycas debaoensis* | KU743927 | 162,094 |
| 5 | *Cycas revoluta* | NC_020319 | 162,489 |
| 6 | *Larix decidua* | NC_016058 | 122,474 |
| 7 | *Pinus koraiensis* | NC_004677 | 117,190 |
| 8 | *Cephalotaxus wilsoniana* | NC_016063 | 136,196 |
| 9 | *Ephedra equisetina* | NC_011954 | 109,518 |
| 10 | *Ephedra foeminea* | KT934791 | 109,584 |
| 11 | *Welwitschia mirabilis* | AP009568 | 118,919 |
| 12 | *Gnetum gnemon* | KP099649 | 115,022 |
| 13 | *Gnetum ula* | AP014923 | 113,249 |
| 14 | *Gnetum montanum* | KC427271 | 115,019 |
| 15 | *Gnetum parvifolium* | NC_011942 | 114,914 |
| 16 | *Selaginella uncinata* | AB197035 | 144,170 |

Table S3 । The codon usage of CP genomes of three *Ephedra* species

| Codon | Count | | | RSCU | | |
| --- | --- | --- | --- | --- | --- | --- |
|  | *E. intermedia* | *E. sinica* | *E. equisetina* | *E. intermedia* | *E. sinica* | *E. equisetina* |
| UUU(F) | 1092 | 1086 | 1096 | 1.47 | 1.47 | 1.47 |
| UUC(F) | 394 | 396 | 394 | 0.53 | 0.53 | 0.53 |
| UUA(L) | 880 | 882 | 880 | 2.2 | 2.2 | 2.2 |
| UUG(L) | 451 | 450 | 450 | 1.13 | 1.12 | 1.13 |
| CUU(L) | 532 | 531 | 531 | 1.33 | 1.33 | 1.33 |
| CUC(L) | 118 | 118 | 118 | 0.29 | 0.29 | 0.29 |
| CUA(L) | 305 | 305 | 305 | 0.76 | 0.76 | 0.76 |
| CUG(L) | 115 | 117 | 116 | 0.29 | 0.29 | 0.29 |
| AUU(I) | 975 | 976 | 977 | 1.52 | 1.52 | 1.52 |
| AUC(I) | 305 | 309 | 305 | 0.47 | 0.48 | 0.47 |
| AUA(I) | 648 | 646 | 647 | 1.01 | 1 | 1.01 |
| AUG(M) | 460 | 459 | 459 | 1 | 1 | 1 |
| GUU(V) | 513 | 513 | 512 | 1.64 | 1.65 | 1.64 |
| GUC(V) | 136 | 133 | 137 | 0.43 | 0.43 | 0.44 |
| GUA(V) | 452 | 446 | 450 | 1.44 | 1.43 | 1.44 |
| GUG(V) | 152 | 152 | 151 | 0.49 | 0.49 | 0.48 |
| UCU(S) | 519 | 515 | 513 | 2.02 | 2.01 | 2 |
| UCC(S) | 182 | 182 | 182 | 0.71 | 0.71 | 0.71 |
| UCA(S) | 278 | 277 | 276 | 1.08 | 1.08 | 1.08 |
| UCG(S) | 149 | 150 | 151 | 0.58 | 0.59 | 0.59 |
| AGU(S) | 333 | 327 | 332 | 1.3 | 1.28 | 1.3 |
| AGC(S) | 81 | 83 | 83 | 0.32 | 0.32 | 0.32 |
| CCU(P) | 428 | 425 | 430 | 1.81 | 1.8 | 1.81 |
| CCC(P) | 122 | 122 | 122 | 0.52 | 0.52 | 0.51 |
| CCA(P) | 284 | 281 | 284 | 1.2 | 1.19 | 1.2 |
| CCG(P) | 113 | 114 | 114 | 0.48 | 0.48 | 0.48 |
| ACU(T) | 495 | 498 | 495 | 1.73 | 1.73 | 1.73 |
| ACC(T) | 154 | 154 | 154 | 0.54 | 0.54 | 0.54 |
| ACA(T) | 383 | 387 | 383 | 1.34 | 1.35 | 1.34 |
| ACG(T) | 111 | 111 | 112 | 0.39 | 0.39 | 0.39 |
| GCU(A) | 572 | 572 | 573 | 1.98 | 1.98 | 1.98 |
| GCC(A) | 139 | 139 | 138 | 0.48 | 0.48 | 0.48 |
| GCA(A) | 332 | 331 | 331 | 1.15 | 1.15 | 1.15 |
| GCG(A) | 113 | 114 | 113 | 0.39 | 0.39 | 0.39 |
| UAU(Y) | 627 | 625 | 626 | 1.65 | 1.64 | 1.65 |
| UAC(Y) | 135 | 138 | 135 | 0.35 | 0.36 | 0.35 |
| CAU(H) | 422 | 421 | 422 | 1.6 | 1.6 | 1.6 |
| CAC(H) | 105 | 104 | 105 | 0.4 | 0.4 | 0.4 |
| CAA(Q) | 805 | 803 | 805 | 1.67 | 1.67 | 1.67 |
| CAG(Q) | 159 | 157 | 159 | 0.33 | 0.33 | 0.33 |
| AAU(N) | 869 | 867 | 871 | 1.52 | 1.52 | 1.53 |
| AAC(N) | 271 | 273 | 270 | 0.48 | 0.48 | 0.47 |
| AAA(K) | 1363 | 1353 | 1361 | 1.6 | 1.6 | 1.6 |
| AAG(K) | 337 | 339 | 338 | 0.4 | 0.4 | 0.4 |
| GAU(D) | 665 | 666 | 666 | 1.52 | 1.53 | 1.52 |
| GAC(D) | 209 | 206 | 208 | 0.48 | 0.47 | 0.48 |
| GAA(E) | 1059 | 1056 | 1060 | 1.63 | 1.63 | 1.63 |
| GAG(E) | 241 | 242 | 240 | 0.37 | 0.37 | 0.37 |
| UGU(C) | 246 | 249 | 247 | 1.6 | 1.61 | 1.6 |
| UGC(C) | 62 | 61 | 61 | 0.4 | 0.39 | 0.4 |
| UGG(W) | 439 | 440 | 439 | 1 | 1 | 1 |
| CGU(R) | 297 | 293 | 297 | 1.36 | 1.34 | 1.36 |
| CGC(R) | 90 | 90 | 89 | 0.41 | 0.41 | 0.41 |
| CGA(R) | 285 | 282 | 285 | 1.3 | 1.29 | 1.31 |
| CGG(R) | 103 | 105 | 103 | 0.47 | 0.48 | 0.47 |
| AGA(R) | 431 | 435 | 430 | 1.97 | 1.99 | 1.97 |
| AGG(R) | 106 | 109 | 106 | 0.48 | 0.5 | 0.49 |
| GGU(G) | 472 | 475 | 471 | 1.44 | 1.45 | 1.44 |
| GGC(G) | 127 | 126 | 127 | 0.39 | 0.38 | 0.39 |
| GGA(G) | 528 | 522 | 527 | 1.61 | 1.59 | 1.61 |
| GGG(G) | 181 | 188 | 182 | 0.55 | 0.57 | 0.56 |
| UAA(*) | 48 | 48 | 48 | 1.97 | 1.97 | 1.97 |
| UAG(*) | 9 | 9 | 9 | 0.37 | 0.37 | 0.37 |
| UGA(*) | 16 | 16 | 16 | 0.66 | 0.66 | 0.66 |
| Total | 23,023 | 22,999 | 23,017 |  |  |  |

* stands for “stop codon”.

Table S4 । SSR types and numbers of CP genomes of three *Ephedra* species

| SSR type | Repeat uint | Amount | | |
| --- | --- | --- | --- | --- |
|  |  | *E. intermedia* | *E. sinica* | *E. equisetina* |
| Mono | A | 21 | 25 | 22 |
| Mono | C | 1 | 2 | - |
| Mono | G | 1 | 1 | 1 |
| Mono | T | 19 | 21 | 20 |
| Di | AT | 2 | 1 | 3 |
| Di | TA | - | 1 | 2 |
| Tri | ATA | 1 | 1 | 1 |
| Tri | TTA | 1 | 1 | 1 |
| Tri | TTC | 1 | 1 | 1 |
| Tetra | AGGT | 1 | 1 | 1 |
| Tetra | ATAA | 2 | 1 | 1 |
| Tetra | ATAG | 1 | 1 | 1 |
| Tetra | CAAA | 1 | 1 | 1 |
| Tetra | CTAC | 1 | 1 | 1 |
| Tetra | TTCT | - | 1 | - |
| Tetra | CTAT | - | - | 1 |
| Penta | ATAAA | 1 | 1 | 1 |
| Penta | TTTTA | 1 | 1 | 1 |
